# INNOVATIVE TECHNOLOGIES: Tobacco Bio-oil Kills Agricultural Pests

**DOI:** 10.1289/ehp.119-a18a

**Published:** 2011-01

**Authors:** Carol Potera

**Affiliations:** **Carol Potera**, based in Montana, has written for *EHP* since 1996. She also writes for *Microbe*, *Genetic Engineering News*, and the *American Journal of Nursing*

Cigarette smoking continues to be the leading cause of preventable death and disease in the United States,^1^ but tobacco has potentially beneficial uses as well as deadly ones. Gardeners have long known that homemade mixtures of tobacco and water can kill insect pests. But these homemade brews kill desirable insects, too, and could poison animals that ingest them. Now researchers at the University of Western Ontario are finding new ways to turn tobacco into a more selective eco-friendly pest control agent.^2^

A team led by chemical engineer Cedric Briens heated finely ground tobacco leaves to 500°C in a vacuum, a process called pyrolysis, then collected the condensate. (Since publishing the paper, the team has found they can use the entire plant—leaves and stalks—which makes it easier and cheaper to harvest the tobacco.) The bio-oil was tested against the Colorado potato beetle (*Leptinotarsa decemlineata*), 11 fungi, and 4 bacteria, all of which are agricultural pests.

The bio-oil blocked the growth of the bacteria *Streptomyces scabies* and *Clavibacter michiganensis* and the fungus *Pythium ultimum. S. scabies* causes a common potato scab disease that makes potatoes unmarketable, *C. michiganensis* kills young plants and deforms fruits, especially tomatoes, and *P. ultimum* kills seedlings of eggplant, peppers, lettuce, tomatoes, and cucumbers. The bio-oil also killed 100% of Colorado potato beetles, a resistant pest that can destroy potato crops. The other organisms were unaffected.

Nicotine, a key toxin in tobacco, has known insecticidal properties on its own. But even after removing nicotine from the bio-oil, it still potently killed these few pests.^2^ The authors say the active components probably include a mixture of phenols with known pesticidal properties working synergistically. They analyzed the bio-oil using gas chromatography–mass spectrometry and note that some of the constituents defy detection. It’s possible new pesticidal molecules are being formed in the high heat conditions of pyrolysis. “We do know that no single molecule is effective, and we seem to have discovered a natural cocktail,” Briens says.

The probable mixture of active chemicals suggests agricultural pests may not readily develop resistance to the bio-oil. Control of the Colorado potato beetle is especially challenging because the beetle is notorious for its ability to adapt rapidly to new pesticides that are applied.^3^ “Insecticides that work now will be obsolete in a few years, and we’ll need new insecticides,” Briens says.

The ability of the bio-oil to target certain agricultural pests could be an asset for future commercialization, because it could spare desirable insects such as honeybees. Some pesticide manufacturers are watching the bio-oil work, but they want to know the active molecules before becoming involved. Then the active components of the bio-oil will require toxicity testing to assess their impact on the environment.

Briens’ study “is a logical and efficient approach to identify a useful by-product of tobacco plants, creating a value-added pesticidal fraction,” says Joel Coats, a professor of entomology and toxicology at Iowa State University in Ames. “The possibility of discovering a novel pesticidal molecule makes the project very worthwhile.”
